# A new hope? Possibilities of therapeutic IgA antibodies in the treatment of inflammatory lung diseases

**DOI:** 10.3389/fimmu.2023.1127339

**Published:** 2023-03-27

**Authors:** Fabian Bohländer

**Affiliations:** Department of Translational Research, Biotest AG, Dreieich, Germany

**Keywords:** IgA, SIgA, mucosal immunity, respiratory disease, immunomodulation, inflammation, immunoglobulin preparation, neutrophils

## Abstract

Inflammatory lung diseases represent a persistent burden for patients and the global healthcare system. The combination of high morbidity, (partially) high mortality and limited innovations in the last decades, have resulted in a great demand for new therapeutics. Are therapeutic IgA antibodies possibly a new hope in the treatment of inflammatory lung diseases? Current research increasingly unravels the elementary functions of IgA as protector against infections and as modulator of overwhelming inflammation. With a focus on IgA, this review describes the pathological alterations in mucosal immunity and how they contribute to chronic inflammation in the most common inflammatory lung diseases. The current knowledge of IgA functions in the circulation, and particularly in the respiratory mucosa, are summarized. The interplay between neutrophils and IgA seems to be key in control of inflammation. In addition, the hurdles and benefits of therapeutic IgA antibodies, as well as the currently known clinically used IgA preparations are described. The data highlighted here, together with upcoming research strategies aiming at circumventing the current pitfalls in IgA research may pave the way for this promising antibody class in the application of inflammatory lung diseases.

## 1 Introduction

With each breath, our airways are exposed to a multitude of inhalable pathogens or toxins. As a contact zone to the environment, with an area of about 100 m^2^, the respiratory tract is a vulnerable part of the human immune defense (1). Therefore, evolution provided this area with a powerful protective machinery, the mucosal immune system. If mucosal immunity is impaired, pathogens can invade and various respiratory illnesses may develop including acute or chronic inflammatory diseases (2, 3).

Respiratory diseases are globally among the most common diseases, especially chronic obstructive pulmonary disease (COPD) and asthma (2). COPD and asthma together with cystic fibrosis (CF) have been identified as the third leading cause of death worldwide (4, 5). Acute respiratory infections can induce pneumonia, either through community- or hospital-acquired pathogens or through inflammation induced by prolonged mechanical ventilation. Particularly severe forms, such as severe community-acquired pneumonia (sCAP), still have a high mortality rates (6–8). Recently, coronavirus disease 2019 (COVID-19) joined the group of respiratory diseases, with high morbidity and mortality especially in patients with risk factors (9).

In the last decades, the number of patients with respiratory diseases has grown, highlighting the need for pharmaceutical interventions (5, 7). Although the medical need in respiratory diseases is high, the probability for a new drug to reach the market has been lower than for other diseases (3% vs. 6-14%). The large diversity and complexity of respiratory diseases paired with limited understanding of the mucosal immune system are aspects that contribute to the challenging development of novel therapies (7).

The mucosal immune system plays a central role in immune surveillance. It is located within mucosal surfaces throughout the body epithelia and protects against infections at the interface to the external environment. This part of the immune system is characterized by a high antibody production to protect against pathogen invasion (10). The immunoglobulin distribution on mucosa differs from that in serum: In serum, IgG is the dominating isotype (75- 80% of serum immunoglobulins), followed by IgA (15%) and IgM (10%) (11, 12). In contrast, on the mucosa, IgA is the predominant class (~74% of all mucosa immunoglobulin) followed by IgG (~25%) and IgM (~2%) (13, 14). The overall production of IgA (40-60 mg/kg per day) is higher than all other isotypes together (15).

IgA has elementary functions in protecting the mucosa from invading pathogens, as well as maintaining homeostasis with the commensal microbiome (16, 17). In humans IgA exits in two subclasses – IgA1 and IgA2 – both are structurally similar but differ in their hinge region and the glycosylation sites. In a recent study Steffen et al. showed the functional relevance of IgA glycosylation. IgA2 induces pro-inflammatory activation of neutrophils and macrophages more potent than IgA1. Accountable for the pro-inflammatory properties of IgA2 were fewer sialic acid glycosylation sites compared to IgA1 (18). The prolonged hinge region of IgA1 makes this subclass more prone to proteolytic degradation, which occurs mainly due to bacterial proteases on the mucosa (19, 20). This is also reflected in the IgA subclass ratio: In serum, IgA1 is dominant (90% IgA1 vs. 10% IgA2), whereas on the mucosa more IgA2 is observed (20-60% of total IgA, depending on the location) (13, 19, 20).

In serum, IgA is mainly found as a monomer with a small portion (~15%) of dimers or other multimers (13, 19, 21). In contrast, mucosal IgA is observed solely in multimeric forms. These forms are covalently linked by the J-chain, a small molecule that facilitates multimerization and is necessary for the binding to the polymeric immunoglobulin receptor (pIgR) and subsequent transport to the mucosa. After translocation, a part of the pIgR, called secretory component (SC), remains attached to IgA and thereby forms the secretory IgA (SIgA) (13, 22). The SC stabilizes the SIgA molecule and protects it from proteolytic degradation (23).

This review provides insights into the current knowledge of IgA, regarding its role in the most common respiratory diseases as well as its functions in infection and inflammation. A special focus will be set on the mucosal immune response. Furthermore, an overview of therapeutically used IgA antibodies and further efforts to use IgA as such in the treatment of inflammatory lung diseases is given.

## 2 IgA in inflammatory lung diseases

Low or absent IgA levels are a relatively common clinical observation, often caused by selective IgA deficiency (sIgAD) the most common type of primary antibody deficiency. Although most of these patients are asymptomatic, the lack of IgA antibodies results in a notable number of patients (~40% of all sIgAD patients) with several immunological disorders (24, 25). The most common disorders, associated with low IgA levels are recurrent respiratory infections, allergic conditions, gastrointestinal disorders or auto-immune diseases (24–30), highlighting the important role of IgA in mucosal areas. Alterations in IgA-mediated mucosal immunity and impaired IgA functions that have been correlated with the most common inflammatory lung diseases are summarized in **Table 1**. The important role of IgA in the mucosal immunity is also demonstrated indirectly by the ability of virulent bacterial strains to produce anti-IgA or anti-FcαRI proteins which help the pathogen to evade IgA-mediated immune responses (26, 51).

**Table 1:** Overview of IgA levels and implications of impaired IgA response in inflammatory lung diseases.

Disease	IgA-levels	Cause of altered SIgA levels	Implication	References
COPD	Serum: reduced BAL: reduced	TGF-β mediated downregulation of pIgR	Higher risk for exacerbations	(1, 2, 31, 32)
Asthma	Serum: reduced BAL: reduced	IL-4 and IL-2 mediated downregulation of pIgR	Reduced immune exclusion of allergens and immunomodulatory effects on DC	(2, 33–36)
CF	Serum: increased BAL: increased	Dysregulation of pIgR expression and dysfunctional SC	Recurrent infections and inflammation	(2, 23, 37)
Pneumonia	Serum: increased or reduced BAL: reduced	Increased consumption, endothelial leakage	Infiltration and inflammatory activation of neutrophils	(38–43)
Severe COVID-19	Serum & BAL: severe course when reduced early and increased in later stages of diseases	Impaired immune response against SARS-CoV-2	Impaired viral clearance from mucosa and enhanced activation of neutrophil inflammation	(44–50)

COPD, chronic obstructive pulmonary disease; SIgA, secretory IgA; BAL, Broncho alveolar lavage; TGF-β, transforming growth factor β; pIgR, polymeric immunoglobulin receptor; IL, Interleukin; DC, Dendritic cells; CF, cystic fibrosis; SC, secretory component; COVID-19, Corona virus diseases 2019; SARS-CoV-2, severe acute respiratory syndrome coronavirus 2.

### 2.1 Chronic respiratory diseases

Chronic respiratory diseases (COPD, asthma and CF) are a group of diseases with similar characteristics, including obstruction of the small airways and episodes of worsening (called exacerbations) due to recurrent airway inflammation (2, 52). Furthermore, alterations of the pIgR/IgA system are described for these diseases [reviewed in (2)].

COPD patients with low serum and/or mucosal IgA levels have a higher risk for exacerbations and recurrent infections (1, 31, 32). The inflammatory environment in the COPD lung is driven by chronically infiltrating neutrophils (1). Inflammation leads to downregulation of pIgR *via* TGF-β and consequently to an impaired IgA transport followed by reduced levels of SIgA on the mucosa (1, 2).

In asthma (type-2 inflammation), the expression of pIgR is downregulated due to IL-4 and IL-13 release by Th2-cells (33). Reduced SIgA on the mucosa leads to decreased immune exclusion of allergens, thereby strengthening the allergic reaction of the immune system. In addition, immunomodulatory effects of SIgA on dendritic cells (DC) and subsequent inhibition of Th2-cell-mediated inflammation are impaired (2, 34).

CF patients suffer from recurrent infections and inflammation, which suggest an impaired mucosal immunity (37). Data regarding the dysregulation of the pIgR/SIgA axis in CF patients are scarce and not fully understood. Due to mutation and misfolding of the epithelial cystic fibrosis transmembrane conductance regulator (CFTR) protein, cellular stress and an unfolded protein response is induced, which inhibits pIgR expression. Concurrently SIgA production and transcytosis was shown to be upregulated through IL-17 induced by chronic *Pseudomonas aeruginosa* infection. In addition increased amounts of dysfunctional SC were observed in sputum of CF patients (2, 23, 37). In summary, the interplay between multiple components affects the pIgR/SIgA axis and induces the dysregulated mucosal immunity in CF.

In COPD, asthma or CF there are multiple alterations and defects affecting the pIgR/IgA axis, contributing to the diseases. A harmful circle of neutrophil-mediated inflammation and impaired SIgA function potentiates chronic inflammation and recurrent infections. The role of neutrophils is dually detrimental in the observed pathology as the released inflammatory mediators can mediate tissue damage, increase inflammation and some, especially neutrophil elastase and proteinase-3, can degrade pIgR, SC and mucosa immunoglobulins (1, 23, 53, 54). Therefore, the regulation of neutrophil inflammation could be a valuable therapeutic strategy.

### 2.2 Pneumonia

Pneumonia is an inflammation of the pulmonary alveoli, which is mostly triggered by infections with bacteria, virus or fungi (55). The most common form of pneumonia is community-acquired pneumonia (CAP), in which the symptom onset occurs in the community (6). Patients with an impaired or dysregulated immune system are at higher risk of developing severe CAP (sCAP) which requires intensive medical care and is associated with high mortality (6, 55).

Besides the persistent infection with highly virulent pathogens, overwhelming inflammation mainly contributes to the pathology of severe pneumonia. The primary immune response induced by the infection in the alveoli facilitates the infiltration of leucocytes from the blood into the alveolar spaces to eliminate the pathogen. However, an overwhelming infiltration and inflammatory activation of neutrophils can trigger tissue damage and more severe lung injury (56, 57). Considering the current challenges and high mortality rates in severe pneumonia, alternative therapeutic approaches, which boost the impaired immune response as well as control the overwhelming neutrophil inflammation, are urgently needed.

An impaired immune response in severe pneumonia is often associated with altered immunoglobulin levels. Diverging data regarding the serum immunoglobulin levels of pneumonia patients were published. Studies showed either lower (38–40) or higher (41, 42) levels of IgA, nevertheless both were associated with an increased mortality risk. Data on alterations in mucosal antibody levels are scarce. A few reports investigated SIgA levels in human bronchoalveolar lavage (BAL) and showed reduced amounts of SIgA in patients with severe pneumonia (43, 58). Interestingly, there seems to be a link between decreased SIgA levels and neutrophil infiltration (43). This could be based on the anti-inflammatory effects of SIgA on neutrophils (59, 60).

### 2.3 COVID-19

Infection with the severe acute respiratory syndrome coronavirus 2 (SARS-CoV-2) induces COVID-19 diseases. While about 20% of people infected with SARS-CoV-2 had a severe form of COVID-19 with pulmonary or systemic inflammation at beginning of the pandemic (61, 62), this has changed to <1% in 2022. Increased vaccination rates and the currently prevalent omicron variants have reduced hospitalization and mortality rates (63, 64). Nevertheless, in patients with an impaired and dysregulated immune response, the infection can still cause inflammation (mainly mediated by neutrophils) and critical damage to the lung (65–67).

The role of serum IgA in COVID-19 has been investigated in early reports. It was shown, that an early and strong serum IgA-response (levels of SARS-CoV-2 specific IgA1 and IgA2) is associated with a mild course of the disease (3, 44–48). Vice versa, decreased serum IgA levels were observed in COVID-19 patients with pneumonia compared to patients with mild course of disease (68), highlighting the importance of antibody-mediated immunity for disease outcome (49, 69, 70). Little information is available regarding the mucosal immunity in COVID-19 (71, 72). It was found that SARS-CoV-2 specific SIgA is an early marker for virus infection and correlates with the severity of disease and inflammatory cytokine response (increase of IFN-β and IFN-γ) (73–75). All these data indicate an elementary role of IgA in mucosal defense against the invading SARS-CoV-2 virus (70, 72). The importance of the mucosal IgA response for the fight against COVID-19 is also reflected in emerging attempts to induce immunization *via* a mucosal route of vaccination (76, 77).

Besides beneficial effects of the mucosal IgA response in protection against SARS-CoV-2 infection, detrimental effects of IgA in COVID-19 have been observed as well. Especially in later stages of COVID-19, when virus neutralizing activity of SIgA gets lost, the outcome can be fatal (49, 75). For example, Staats et al. describes the correlation of anti-SARS-CoV-2 IgA antibodies in serum with neutrophil extracellular trap (NET) formation in severe COVID-19 cases. By measuring SARS-CoV-2 specific IgA- and IgG levels, CRP and extracellular DNA, they found that especially antibodies of IgA2-subclass are potent activators of neutrophil inflammation and NET-formation (78). The IgA-mediated enhancement of NET-release was also reported by others (79–81). In COVID-19 enhanced NET-formation is correlated to a fatal outcome, as reported by several groups (49, 82–85). Therefore LaSalle et al. hypothesized that early IgA induced NET-release is beneficial in the mucosal areas to prevent SARS-CoV-2 entry, whereas NET-release in later stages can be harmful in the circulation and promote tissue damage (49). This represents an important aspect for the development and application of IgA-based COVID-19 therapies.

### 2.4 IgA and neutrophils

IgA is able to interact with several Fc-receptor expressing immune cells like neutrophils, eosinophils, monocytes or Kupffer cells. Furthermore IgA can interact by binding to alternative IgA receptors (like DC-SIGN, transferrin receptor or FcRL4) with, for example, T-cells or dendritic cells. These interactions can activate immune cells (e.g. enhance the phagocytosis of pathogens by macrophages) or induce potent immunomodulation (e.g. the expansion of regulatory T-cells) (86, 87).

However, when looking at the above mentioned inflammatory lung diseases, the interaction between IgA and neutrophils is from particular interest. Based on their large number (50-70% of human leucocytes) and potent cytotoxic capabilities (e.g. degranulation, oxidative burst, NETs, or proteases) neutrophils play a key role in promoting chronic inflammation, tissue damage and overall diseases pathogenesis (88). The interaction of IgA with the neutrophil IgA-Fc-receptor (FcαRI) is able to activate a strong intracellular signaling cascade (see chapter 3 below), which is capable to induce the release of leukotriene B4 (LTB4). LTB4 is a potent neutrophil chemoattractant, which leads to the infiltration of large numbers of neutrophils into the lung. The self-containing positive feedback loop of IgA-mediated activation and neutrophil infiltration can induce overwhelming neutrophil response with detrimental effects on the lung tissue (86, 89).

Therefore and although IgA interacts with several other immune cell types (like monocytes, DC or T-cells) the interaction with neutrophils is the most relevant in view of inflammatory lung diseases.

## 3 The functional role of IgA

As highlighted in the previous chapter, IgA immunity is impaired in the most prevalent inflammatory lung diseases. This indicates that IgA antibodies have important functional roles, in serum and on the mucosa. In the following, an overview of the currently known IgA functions is given.

Based on binding to the FcαRI, IgA can mediate a dual role in immunity. On the one hand, IgA has anti-pathogenic properties protecting against infections and on the other hand, IgA has potent immunomodulatory functions thereby maintaining immune homeostasis (87, 89–91).

Multivalent binding of IgA, within an immune complex, induces FcαRI cross-linking, which activates the Src family kinase Fyn. This kinase fully phosphorylates several immunoreceptor tyrosine-based activation motifs (ITAM) leading to recruitment of the kinase SYK. This kinase activates several downstream kinases and signaling pathways, ultimately leading to activation of MAP kinase and NF-κB, which results in pro-inflammatory cell activation (90, 92).

In contrast, monovalent binding of IgA lead to recruitment of Src family kinase Lyn. In contrast to Fyn, Lyn phosphorylates only a single tyrosine motif, which induces the recruitment and activation of SHP-1 phosphatase. SHP-1 inhibits pro-inflammatory responses from activating Fc- or toll-like-receptors by formation of inhibisomes. This inhibitory ITAM (ITAMi)-signaling results in cellular immunomodulation (90, 93).

Noteworthy is also a third way of Fc-receptor signaling, the immunoreceptor tyrosine-based inhibition motif (ITIM) signaling, which is solely induced by the inhibitory IgG-FcγRIIB (90, 92).

### 3.1 Function of IgA in serum

#### 3.1.1 During infection

As described above the functions mediated by serum IgA are dependent on the interaction with FcαRI. In case of infection IgA-antibodies recognize and opsonize invading pathogens and their toxins. Binding of multivalent IgA-immune complex to FcαRI induces – depending on the immune effector cell type – several pro-inflammatory effector functions. These include firstly direct clearance mechanism like neutralization, phagocytosis, degranulation, NET formation, release of reactive oxygen species (ROS) or antibody dependent cellular cytotoxicity (ADCC). And secondly the further activation of the immune response, for example due to the release of inflammatory cytokines or chemokines, antigen presentation or the recruitment of neutrophils by the chemoattractant LTB4 [reviewed in (13, 51, 86, 87)].

Independent of FcαRI, IgA has the ability to induce the alternative and lectin pathway of the complement system, thereby promoting complement dependent pathogen clearance (94).

#### 3.1.2 During inflammation

In contrast, and in the absence of any pathogenic antigen, monomeric serum IgA mediates important immunomodulatory functions *via* FcαRI and ITAMi signaling, thereby restoring a homeostatic state after inflammation. It was shown that IgA downregulates several inflammatory cell responses, e.g. IgG-mediated phagocytosis, chemotaxis, oxidative burst and inflammatory cytokine release [reviewed in (87, 91, 95)]. Furthermore, IgA blocks IgG-mediated complement activation (96) and counteracts IgE-FcϵRI-induced mast cell degranulation (97, 98). ITAMi signaling is therefore described as a critical mechanism to maintain immune homeostasis (92, 98).

Apart from FcαRI and innate immune cells, it was shown that IgA is able to modulate the T-cell response. Saha et al. demonstrated that monomeric serum IgA is able to inhibit Th17-cell mediated inflammation, and concurrently expand FoxP3^+^ Treg-cells *via* F(ab’)_2_ binding to cytokine receptors (99).

### 3.2 Function of IgA on lung mucosa

**Figure 1:**
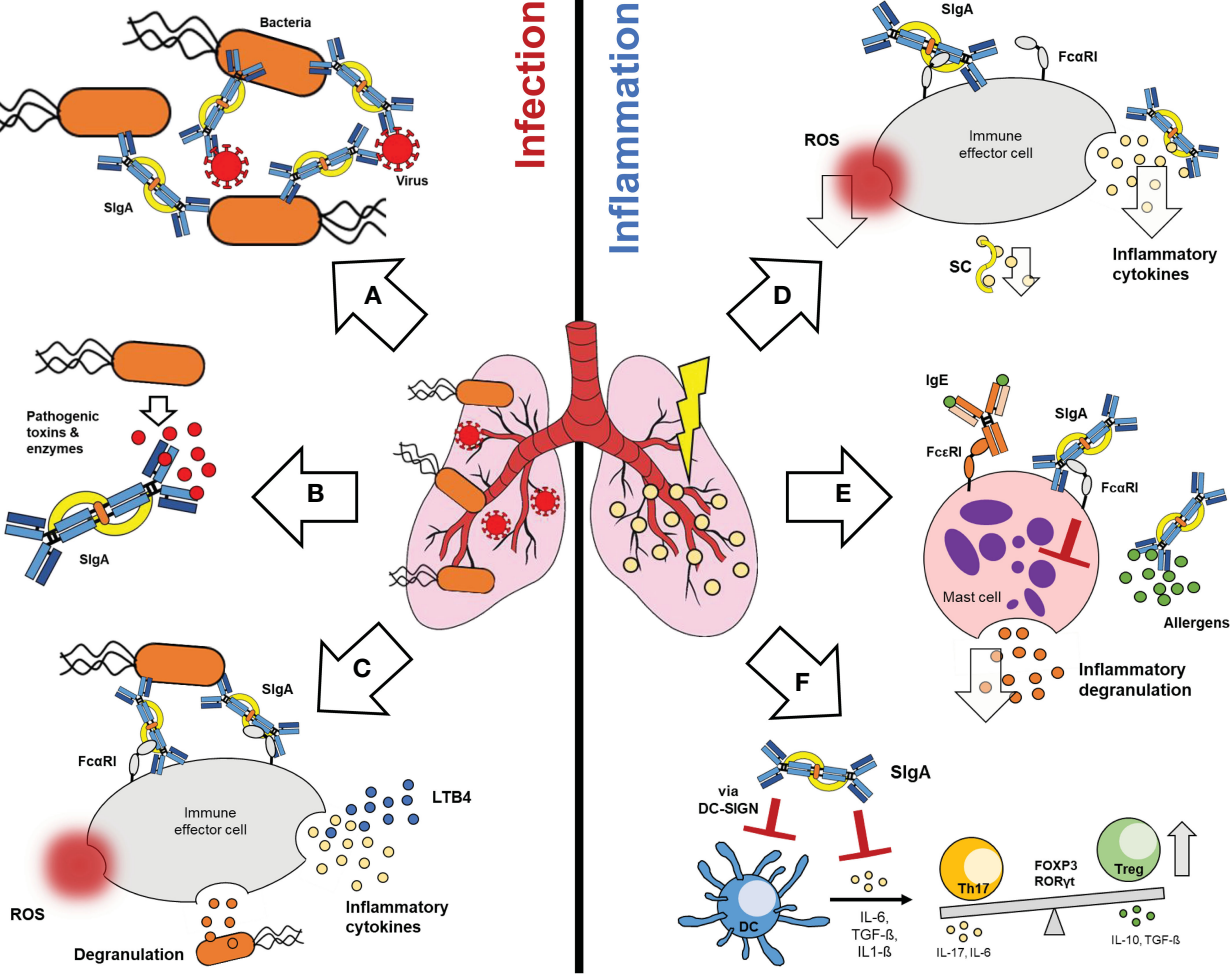
Functions of SIgA in lung infection and inflammation. During infection, SIgA induces potent anti-pathogenic effects (left): **(A)** SIgA can efficiently agglutinate bacteria and virus, **(B)** neutralize pathogenic toxins and enzymes and **(C)** activate FcαRI dependent inflammatory effector functions on immune effector cells. These include ROS generation, degranulation or the release of inflammatory cytokines. Furthermore, SIgA-FcαRI cross-linking on neutrophils mediates the release of LTB4, which leads to recruitment of neutrophils to the lung. On the other hand, SIgA can induce various immunomodulatory effects during lung inflammation (right): Binding of SIgA to FcαRI on immune effector cells can reduce the release of ROS and inflammatory cytokines. SC alone can reduce inflammatory cytokines **(D)**. In case of allergic inflammation, SIgA can prevent binding of allergens to the mucosa and reduces FcεRI dependent degranulation of mast cells via FcαRI binding **(E)**. SIgA can also interact via DC-SIGN with dendritic cells, thereby modulating inflammatory cytokine release and T-cell responses **(F)**. SIgA, Secretory Immunoglobulin A; FcαRI, Fc alpha-receptor I; ROS, Reactive oxygen species; LTB4, leukotriene B4; SC, secretory component; IgE, Immunoglobulin E; FcεRI, Fc epsilon-receptor I; DC-SIGN, Dendritic cell-specific ICAM-3-grabbing non integrin; DC, Dendritic cell; Th17, T-helper 17 cell; FOXP3, Forkhead-Box-Protein P3; RORγt, RAR-related orphan receptor gamma-t; Treg, T-regulatory cell.

The modes of action proposed for SIgA antibodies in lung infection and inflammation are summarized in **Figure 1**.

#### 3.2.1 During infection

The main function of SIgA on the lung mucosa is to provide protection against invading pathogens. This is possible due to its unique properties, which include high valency binding to pathogens (due to its multimeric structure) and the high resistance to bacterial proteases (due to SC) (1, 100).

During infection SIgA induces agglutination of bacteria and virus, thereby preventing pathogen binding to lung epithelium (**Figure 1A**). Agglutinated pathogens are rapidly cleared through the mucus, a process known as immune exclusion. The fact that pIgR can transport IgA alone or in complex with an antigen, allows the efficient intracellular neutralization of virus within infected epithelial cells during transport to the mucosa. In addition, pathogens that invaded into the lamina propria can be opsonized by dimeric IgA and transported back to the mucosa, a process called antigen excretion. In addition to pathogens, SIgA can also efficiently neutralize pathogenic toxins and enzymes (**Figure 1B**) (13, 16, 17, 87). The importance of IgA multimerization for efficient pathogen binding and neutralization was demonstrated several times and has to be highlighted as a central element in therapeutic IgA antibody development (101–104).

Like serum IgA, activation of FcαRI bearing effector leucocytes *via* SIgA has been reported, although binding of SIgA to FcαRI is partially hampered due to steric hindrance of SC (**Figure 1C**) (51, 87, 89, 105). Based on transgenic mouse and *in vitro* studies, SIgA binding can promote inflammatory activation of the mucosal immune response e.g. phagocytosis, ADCC, ROS generation, degranulation and inflammatory cytokine secretion (1, 2, 23, 51, 74). Furthermore, the binding of SIgA opsonized pathogens to FcαRI on neutrophils, induces LTB4-release and recruitment of further neutrophils to the lung (**Figure 1C**) (87, 89).

#### 3.2.2 During inflammation

Despite the generally well-known anti-pathogenic functions, SIgA mediates also important immunomodulatory effects on different mucosal immune cell types. Similar to monomeric serum IgA, it is proposed that the low affinity binding of SIgA to FcαRI, in absence of antigen, leads to immunomodulatory effects *via* inhibitory ITAMi signaling on mucosal effector leukocytes (**Figure 1D**) (2, 74, 89).

The anti-inflammatory effects of SIgA were frequently demonstrated by looking at the cytokine response. Under homeostatic conditions SIgA can reduce the pathogen-mediated inflammatory cytokine response, downregulates the oxidative burst and the release of inflammatory cytokines of neutrophils and epithelial cells *in vitro* (**Figure 1D**) (59, 60, 97, 106, 107). However, not only SIgA but also the SC alone has immunomodulatory properties. It was shown that the SC can neutralize IL-8, an important chemokine for neutrophil migration (**Figure 1D**) (108).

In addition to infection-mediated inflammation, SIgA has important anti-inflammatory functions in context of allergic reactions. Due to immune exclusion SIgA prevents allergen binding and activation of the mucosal immune response (2, 97), as well as IgE-mediated degranulation of mast cells *via* cross-linking of FcαRI (**Figure 1E**) (97, 98).

Furthermore, cells of the adaptive immune response can be modulated by SIgA. It was shown that SIgA interacts with DC in mice *via* SIGNR-1 (the human analog to DC-SIGN), thereby inhibiting pro-inflammatory cytokine production and inducing IL-10 production *via* expansion of regulatory FoxP3^+^ Treg-cells (**Figure 1F**) (34, 91).

## 4 IgA as therapeutic antibody

IgA is a crucial player in the immune defense as well as in control of overwhelming inflammation. When the functions of mucosal SIgA are impaired or IgA levels are low, respiratory diseases can develop (1, 2, 33, 109–111). Which represents the rationale for the development of therapeutic IgA antibodies.

### 4.1 Hurdles and benefits with IgA as therapeutic antibody

With a few exceptions, almost all therapeutically used antibodies (recombinant or plasma-derived) are of IgG class (112). This can be attributed to the natural abundance of this antibody class in the human body, the long half-life, but also to the comprehensive knowledge about IgG and the limited knowledge about the other isotypes (113). The research and clinical use of IgA was historically impaired due to several reasons: (i) Issues with small animal models, especially rodents, hamper the research with IgA. Rodents and humans have major physiological differences. Mice have, like most other species, one subclass of IgA, which is dimeric in serum. In contrast, human IgA exists in two subclasses and is mainly monomeric, a feature shared only with chimpanzees, gorillas and gibbons (114–116). The major IgA receptor FcαRI is missing in rodents (19, 51, 117, 118) and human IgA has only a short half-life in mice (119). (ii) Problems with recombinant production of IgA. Because IgA is highly and heterogeneously glycosylated, the production can cause altered glycosylation patterns and therefore enhance immunogenicity and clearance (19, 105, 112). (iii) The short half-life in humans compared to IgG which would require shorter dosing intervals (13, 27, 120).

Although IgA antibodies were often not considered in research, they have some major advantages, e.g. IgA can activate more potent cellular effector functions in comparison to other immunoglobulin isotypes. Comparison of IgG and IgA functions was mainly done in the context of cancer research, because IgG lacks efficient activation of neutrophils (113). It has been shown that IgA, *via* FcαRI, induces a much stronger neutrophil-mediated tumor cell killing than IgG. Mechanistically this could be reasoned through enhanced migration of neutrophils, induced by IgA mediated LTB4 release. Furthermore, IgA induces stronger phagocytosis by macrophages as well as ADCC, oxidative burst, cytokine and NET release by neutrophils (19, 119–121). A possible explanation therefore is, that although FcαRI expression is lower than FcγR expression, IgA-FcαRI binding induces stronger ITAM signaling than IgG-FcγR binding, which was found to be due to the 2:1 stoichiometry in IgA-FcαRI binding (113, 122).

Apart from the stronger immune activation by IgA-FcαRI ITAM-signaling, IgA can also mediate stronger immunomodulation *via* inhibitory ITAMi pathway compared and counteracting to IgG. Which was demonstrated using a neutrophil cell-line (123, 124). These immunomodulatory properties have a great therapeutic potential in the treatment of inflammatory diseases (95, 105).

### 4.2 Overview of clinically used IgA antibodies

Currently only plasma-derived IgA antibodies are therapeutically used. Plasma-derived IgA can be received either directly from plasma donations (e.g. convalescent plasma or fresh frozen plasma) or from purified antibody preparations (100, 125). For the latter, antibodies were purified from a pool of thousands of plasma donations from healthy donors. Such preparations are known since the 1950s as intravenous immunoglobulin preparations (IVIg) for the treatment of antibody deficiencies and nowadays additionally for the treatment of a plethora of autoimmune- and inflammatory-diseases (100, 126). As plasma-derived antibodies are from human origin and usually not modified during the manufacturing process, they were well tolerated and safe (127). The high diversity in these polyclonal preparations allows it to target a multitude of pathogens and induce several modes of action simultaneously (127–129).

In contrast to the standard IVIg, which contains >95% IgG (130), there are four preparations with notable amounts of IgA used in clinical settings so far: IgAbulin, Venimmun, Pentaglobin and trimodulin (12, 27, 100, 126) (**Table 2**).

**Table 2:** Overview of clinically used plasma-derived antibody preparations containing notable amounts of IgA.

Product and manufacturer	Ig-distribution*	Indication and administration	Development status	References
**IgAbulin** Immuno AG	73% IgA 26% IgG 1% IgM	Oral or nasal application. Clinical studies in NEC and treatment/prevention of respiratory tract infections.	Produced for experimental clinical use only	(126, 131–137)
**Venimmun N** CSL Behring GmbH	12% IgA 80% IgG 8% IgM	For IV use. Clinically tested as immunomodulatory agent in ITP and Crohn’s diseases, further as substitution therapy in antibody deficiencies.	Produced for experimental clinical use only	(12, 126, 138–140)
**Pentaglobin** Biotest AG	12% IgA 76% IgG 12% IgM	For IV use. Approved for the adjunctive treatment of bacterial infections and immunoglobulin substitution in patients with immunodeficiency or severe secondary antibody deficiency syndrome.	Marketed product	(126, 141–143)
**Trimodulin** (BT588 & predecessor BT086) Biotest AG	21% IgA 56% IgG 23% IgM	For IV use. In development as adjunctive treatment to standard of care in sCAP and COVID-19.	Clinical phase III in COVID-19 (NCT05531149) and sCAP (NCT05722938) ongoing	(144–147)

Immunoglobulin composition, development status and current usage were compared. *Ig distribution are approximate values. IV, intravenous; NEC, necrotizing enterocolitis; ITP, immune thrombocytopenic purpura; sCAP, severe community acquired pneumonia; COVID-19, Coronavirus diseases 2019.

#### 4.2.1 IgAbulin

IgAbulin was a plasma-derived preparation consisting mainly of IgA (~70% of Ig’s), but also notable amounts of IgG (~30% of Ig’s) and additionally transferrin (~10% of total protein). Approximately 74% of the total immunoglobulin was monomeric, ~17% dimeric, ~1% polymeric and ~3% fragmented (126, 131, 132).

The anti-inflammatory effects of IgAbulin were investigated *in vitro*. IgAbulin downregulated FcαRI-dependent the release of TNF-α and IL-6, as well as the oxidative burst of neutrophils and monocytes. In contrast the release of the inhibitory IL1-ra was enhanced (107, 148, 149). IgAbulin demonstrated more potent anti-pathogenic properties than IVIg, by neutralizing streptococcal superantigens *in vitro* (150).

In a double blind, placebo-controlled study, IgAbulin was administered as a nasal spray to children with rhinitis. Children in the IgAbulin group had significant reduced days with rhinitis compared with placebo (*n=40, p=0.004*) and the ease of use was convincing (133). In another placebo-controlled trial in children with frequent respiratory infections, IgAbulin was effective in the prophylaxis and reduction of upper respiratory tract infections (*n=36, p < 0.012*). In this study IgAbulin was applied *via* nose drops in the nasal cavity and showed a good safety profile, as well as a convenient application to the children (134).

In two similar experimental studies, IgAbulin was used as prophylaxis to prevent infections in adult top-athletes. The nasal application lead to an increase in nasal salvia IgA levels, however reduced upper respiratory tract infections only in one of the studies significantly (*n=34, p < 0.01*) (135, 136).

Besides intranasal administration, the efficacy of IgAbulin was also tested after oral administration. In a prospective study of infants and children with chronic diarrhea IgAbulin lead to a reduced number of stools per day (*n=7, p < 0.05*) (131) and in a randomized clinical trial prevents necrotizing enterocolitis (NEC) of preterm infants and neonates (*n=179, p=0.0143*) (132). However, a meta-analysis concluded no significant benefit after oral IgAbulin administration, which does not support the further use of IgAbulin in NEC (137).

The monomeric form of IgA, which was the main ingredient, could be a reason for the limited efficacy of IgAbulin (100). However, and importantly, no concerns with anaphylactic reaction caused by pre-existing anti-IgA antibodies were observed, which was for a long time considered as a possible risk factor in IgA therapy (126, 151).

The usage of IgAbulin was safe and well-tolerated justifying the use of therapeutic IgA preparations. The application of IgAbulin to prevent respiratory tract infections is the only known clinical delivery of a therapeutic IgA antibody to the mucosa and therefore a potential starting point for further approaches. Using multimeric-polyvalent IgA preparations or higher dosage could be more effective and might be an option to be further investigated in the future.

#### 4.2.2 Venimmun N

Venimmun N was a plasma preparation, with an IgA-portion of ~12%, but there are no data about the biochemical structure of these IgA molecules available (126). Venimmun N was used in experimental studies as substitution therapy in patients with immune deficiencies and as prophylaxis for septic complications, with no clear effects (138, 152, 153).

In the treatment of autoimmune diseases, Venimmun N showed contradicting results. Although in case studies the treatment of patients with Crohn’s diseases mediated beneficial immunomodulatory effects, there was no benefit compared to a standard IVIg (139, 154). Moreover, in a prospective crossover trial with 177 immune thrombocytopenic purpura (ITP) patients no significant differences in the response rates of Venimmun N treatment could be demonstrated (140). In a comparative *in vitro* study, Venimmun showed stronger modulation of TNF-α release than standard IVIg in microglia cell culture. An effect partially attributed to the IgA component. However, another IgM/IgA-enriched preparation Pentaglobin showed much more potent effects on TNF-α release and nitric oxide production (155).

In most of the mentioned studies, Venimmun N was considered as a pure IgG preparation; therefore, no evaluation of the therapeutic effect of IgA in this preparation can be performed.

#### 4.2.3 Pentaglobin

Pentaglobin is a human plasma-derived immunoglobulin preparation containing IgM, IgA and IgG. The immunoglobulin distribution in Pentaglobin (~12% IgM, ~12% IgA and ~76% IgG) is comparable to that in human serum (12, 126). Biochemical characterization of Pentaglobin showed a complex mixture of monomeric IgG, monomeric IgA, dimeric and trimeric IgA, as well as pentameric IgM. The multimeric IgA and IgM species were shown to be associated with notable amounts of J-chain, which could enable the transport to mucosa and support the usage in inflammatory lung diseases (156).

Indeed, in a case report of a common variable immunodeficiency patient it was shown that IgA applied with Pentaglobin can be transported onto the lung and help to reduce exacerbations of bronchiectasis (157, 158). Furthermore, the IgA portion of Pentaglobin was shown to be transported to the lung of rats and mice, with notable amounts of functional active IgA present in the BAL (159, 160). The presence of administered immunoglobulins on the lung helped to defeat bacterial infections and protected lung tissue *via* immunomodulatory effects in a mouse model of stroke associated pneumonia (160).

In focus of systemic inflammatory diseases, like sepsis and septic shock, the use of plasma-derived immunoglobulin preparations is a promising approach, as these can mediate both – pro- and anti-inflammatory – effector functions (161). These dual functions were demonstrated for Pentaglobin in various *in vitro* and *in vivo* studies.

The anti-pathogenic functions of Pentaglobin comprise the opsonization and phagocytosis of pathogens (162–164), as well as the neutralization of bacterial endo- and exotoxins (164–166). Functions, which were mediated more efficiently by Pentaglobin in comparison to standard IVIg (167–169). It was shown that all immunoglobulin species (IgM, IgA and IgG) within Pentaglobin have titers against surface antigens of the most common pathogens involved in sepsis (170).

Pentaglobin showed stronger immunomodulatory properties than IVIg, like the modulation of complement factors (171–173) and cytokines (e.g. IFN-γ, TNF-α, IL-6, IL-8, IL-1β, IL-10) (167–169, 174). Furthermore, Pentaglobin demonstrated immunomodulatory effects on T-cells *in vitro* and *in vivo* (168, 175, 176).

In most of the mentioned studies these effects were attributed to the IgM component, thereby not adequately addressing the role of IgA in this polyclonal preparation (126). However, as pure IgM and IgA controls were lacking in these studies, some effects could also be attributed to IgA. Recent data confirm that IgA can have important effector functions in such IgM/A-enriched immunoglobulin preparations (123).

Apart from the preclinical data, there is a lot of clinical experience gathered in the last decades with Pentaglobin. Pentaglobin was approved in 1985 (177) for the use as adjuvant therapy of severe bacterial infections and immunoglobulin substitution in immunocompromised patients.

In septic patients, the levels of the main immunoglobulin isotypes IgM, IgA and IgG are often decreased and were associated with a reduced survival rate (178, 179). Therefore, the supplementation of all three classes seems to be a promising strategy (161). Pentaglobin has been investigated in numerous clinical trials for the adjunctive therapy of sepsis in adult (141–143) and in several trials in paediatric patients and neonates with sepsis (180–183). As mentioned in several reviews the additional IgM- and IgA-components in Pentaglobin mediate beneficial effects in the treatment of inflammatory diseases compared to IVIg (100, 126, 151, 161, 177, 184). This was confirmed in systematic meta-analyses, where a significant reduction of mortality and length of mechanical ventilation was shown after Pentaglobin treatment in patients with severe sepsis and septic shock (141–143). This effect was not that pronounced after IVIg treatment (141, 143).

Furthermore, there were some case reports and small studies regarding the usage of Pentaglobin as adjunctive therapy in COVID-19 (185–188). Although in a large retrospective cohort study, no significant difference in mortality was observed (*n=316, p=0.374*), a subgroup of severe COVID-19 patients who have not yet received invasive mechanical ventilation may benefit from Pentaglobin therapy (*p=0.063*). It has been observed that an early and high dose of Pentaglobin seems to help patients with low IgM, IgA and IgG levels (189).

The efficacy and safety of Pentaglobin was shown in various studies and countless regular administrations over decades, therefore Pentaglobin is currently the longest lasting and best characterized therapeutically used preparation containing IgA. Simultaneously there is limited knowledge regarding the functional role of IgA in Pentaglobin. Based on the complexity and heterogeneity of this preparation containing IgM, IgA and IgG, it is challenging to attribute beneficial effects to a distinct component. With emerging data regarding the immunoglobulin biochemistry, it seems to be feasible that these polyclonal immunoglobulin preparations could also mediate protective effects in inflammatory lung diseases, where especially multimeric IgA species could be an object of interest.

#### 4.2.4 Trimodulin

Trimodulin is a human plasma-derived native polyvalent antibody preparation for intravenous administration with ~23% IgM, ~21% IgA and ~56% IgG. Based on a new manufacturing process, native immunoglobulins with more active Fc- and Fab-binding sites compared to Pentaglobin were purified.

Trimodulin is in clinical development for the treatment of sCAP and moderate/severe COVID-19, two inflammatory lung diseases. In a *post-hoc* analysis of a phase II trial (NCT01420744) conducted in sCAP patients, trimodulin showed significant reduction in mortality in a subgroup of invasively ventilated patients with low IgM (≤ 0.8 g/L) and/or high CRP (≥ 70 mg/L) levels (*n=92, p=0.006*). Furthermore trimodulin prevents secondary infections, based on a reduced number of infection-related adverse events (144). During the recent pandemic, the development of trimodulin was expanded to the additional indication, severe COVID-19. A phase II study in patients with severe COVID-19 [NCT04576728 (145)] showed reduced mortality in a subgroup of hospitalized patients with early systemic inflammation (*manuscript under preparation*). Based on the promising results from the two phase II trials, development of trimodulin now continues in two phase III trials in sCAP [NCT05722938 (190)] and moderate/severe COVID-19-patients [NCT05531149 (146)].

The comprehensive preclinical characterization identified three relevant modes of action for trimodulin: (i) opsonization and clearance of pathogens, (ii) neutralization of toxins and (iii) modulation of the inflammatory response.

The opsonization and clearance of causal pathogens was shown by a large repertoire of antigen binding activities, as IgM, IgA and IgG antibody titers against relevant bacteria, viruses and fungi were present in trimodulin. It was shown that the antibodies of trimodulin can opsonize and enhance phagocytosis *in vitro* (123, 191). The trimodulin mediated phagocytosis and inflammatory activation of neutrophils was shown to be dependent on IgA-FcαRI and IgG-FcγR binding, highlighting the anti-pathogenic effects of IgA in this therapeutic antibody preparation (123).

The efficient neutralization of bacterial toxins by IgM, IgA and IgG plasma preparations is known from literature (150, 165–169), but was also demonstrated for trimodulin. Trimodulin can effectively neutralize pneumolysin and protect platelets from pneumolysin-induced damage (192, 193).

The immunomodulatory properties of trimodulin on the release of several cytokines and chemokines (e.g. TNF-α, IL-17a, IFN-γ, MCP-1, IL-8, IL-1ra and IL-10) were assessed in different cell-culture models and shown to be significantly stronger compared to IVIg (123, 124, 194). In a neutrophil-cell model, the endotoxin-induced IL-8 release was reduced *via* binding of the trimodulin IgA-component to FcαRI and subsequent ITAMi signaling (123). As the FcR-mediated effects of IgM on neutrophils are of minor importance, the beneficial effects over IVIg can be attributed to the IgA component (123, 124). Furthermore, direct binding and neutralization of IL-8 by trimodulin was more potent than by IVIg, an effect that can be related to the multimeric IgM and IgA species (123).

Immunomodulation by trimodulin was also observed on the expression of monocyte and neutrophil cell surface receptors, like FcR, TLR and complement receptors (123, 194). On the neutrophil-like HL-60 cell-line, trimodulin counteracted the endotoxin-mediated upregulation of activating FcR for IgG (FcγRI, FcγRIIA and FcγRIII) as well as IgA (FcαRI). Effects which were not observed due to IVIg treatment (123). These modulatory effects on surface receptor expression seem to counteract inflammatory cell responses, like phagocytosis, ADCC or chemokine release. The altered activation of intracellular signaling cascades *via* IgA-FcαRI binding could to be key in modulating the neutrophil inflammatory response.

Furthermore, trimodulin has a dual and balancing function on the complement system. By either activating complement and induce opsonophagocytosis, or avoiding excessive activation by interacting with the activated complement factors C3b and C4b (191). This balancing effect on anaphylatoxins were also observed in healthy subjects and sCAP patients treated with trimodulin (144, 191).

The data regarding the immunomodulatory modes of action of trimodulin give a complex picture. The antibody isotypes target several receptors and mechanism simultaneously, leading to more potent immunomodulation compared to a pure IgG preparation. It is difficult to attribute the pharmacological effects to one immunoglobulin component; rather it is feasible that all components together (IgM, IgA and IgG) mediate the observed clinical effects, as mentioned by others (179). Nevertheless, it was clearly shown that the (often neglected) IgA component of trimodulin mediates powerful effects, especially *via* FcαRI binding on neutrophils (123, 124).

Based on the high IgA content and the potent immunomodulatory activity, trimodulin is a promising preparation for the treatment of inflammatory lung diseases. Further research is necessary to expand the knowledge regarding the role of IgA in this preparation and the intended indications, as this may help to take advantage of the full therapeutic potential of this product.

### 4.3 Upcoming strategies using IgA in respiratory diseases

#### 4.3.1 Application of IgA to the lung

The direct application of therapeutic IgA antibodies onto the lung offers a great opportunity compared to systemic delivery as it can support the respiratory immune defense before pathogens enter the circulation, which was shown in several animal models (195–202), or protect the lung tissue by anti-inflammatory effects as shown in mice (106, 197, 203, 204). Inhalation ensures a rapid and high amount of drug directly delivered to the site of action in the lung, which is not achieved with other administration forms. Therefore, a lower dose can be used to achieve the same efficacy, which avoids possible side effects (205).

Technically, antibodies can be delivered to the lung *via* metered dose inhalers, dry powder inhalers or nebulizers (206). These oral inhalation procedures should be distinguished from intranasal administration (e.g. *via* nasal sprays), as the latter applies the drug to the nasal mucosa, whereas oral inhalation reaches the lung mucosa (7). Inhalation therapy is routinely used in the treatment of chronic lung diseases, e.g. asthma or COPD. Administration by inhalation allows self-administration and is more comfortable to the patient in comparison to invasive administration with a needle (205, 207).

Some monoclonal antibodies applied *via* inhalation route are currently in preclinical or clinical trials (205, 207). Furthermore, some clinical tests were performed with inhalable IVIg (208, 209). However, development of antibodies for administration by inhalation is challenging and the failure rate high. Firstly, the pre-clinical testing in animal models is difficult as rodents are obligate nose breathers and have several anatomical differences compared to humans, which lead to different distribution and clearance patterns (7, 210). Secondly, the pharmacokinetics of inhaled antibodies are not as favorable, because the half-life is with ~ 1 day (for IgG) much shorter than in human serum (~23 days) (205, 211). Thirdly, stability during the nebulization process can be critical for the development, as immunogenic aggregates can be formed (212).

To circumvent these issues, there are some approaches to enhance stability of proteins during nebulization process and enhance residence time in the lung. These approaches include protein engineering, formulation- and carrier development, or optimization of the nebulization process (212).

Antibody engineering approaches aim to modify the structure of antibodies to enhance lung residence time and stability. These include the generation of smaller antibody fragments, Fc-engineering or PEGylation (213). Conjugation of polyethylene glycol (PEG) chains to proteins increases the molecular size and mucoadhesion, thereby leading to enhanced resistance against proteolytic digestion and alveolar uptake (213–215). The development of formulations and carrier particles specifically for inhalation therapy offers a variety of solutions to prolong residence in the lung. Besides the classical additives (e.g. salts, sugars or surfactants), several carrier-formulations like liposomes, micelles, nano- or micro-particles and microspheres were developed (216). Based on particle size/shape and surface charge/coating the deposition, retention and cellular uptake of carrier particles can be influenced (205, 217, 218). During the nebulization process it is crucial to maintain the molecular integrity and potency of the drug, as well as targeted delivery. These critical parameters are driven by the nebulization device and the particle size (213, 217).

SIgA has an outstanding role in the mucosal immunity and its function is impaired in several inflammatory lung diseases, as mentioned within this review. Therefore, supplementation with inhalable therapeutic IgA antibodies might be a worthwhile strategy (2). Especially with regard to neutrophil-mediated inflammation, modulation by IgA seems to be promising (1, 23, 123, 124).

However, only a limited number of monoclonal IgA antibodies were used for the intranasal delivery to the respiratory system in animals (219, 220) and despite promising results did not reach clinical testing. To avoid further setbacks, e.g. an excess of therapeutic IgA might be applied, as degradation of IgA by neutrophil- and bacterial proteases could have hampered clinical effects in previous approaches. Alternatively, IgA preparations could be improved in a manner that mimics SIgA structural stability, to be efficiently used on the mucosa (27).

Hence, Longet et al. coupled a recombinant SC to plasma-derived multimeric IgA and IgM, thereby creating secretory like molecules (21, 106). *In vitro* these SIgA and SIgM molecules were less prone to proteases and protected epithelial cells from bacterial-induced damage (21, 106). In three *in vivo* mouse models SIgA/IgM, as well as other plasma-preparations (monomeric IgG (IVIg), monomeric IgA and multimeric IgA/M) were applied orally or intranasally. The immunoglobulins reduced mucosal infection and protected the animals from lethal bacterial and viral infection. In contrast to the *in vitro* data, coupling of the SC to IgA/M was not beneficial and IVIg was more efficient *in vivo* (195, 197, 202). As part of one study, the immunoglobulins were nebulized and administered *via* oral inhalation to rats and non-human primates. IgM, IgA and IgG remained structurally and functionally intact during the nebulization and were detectable in the BAL of these animals (202). In addition to the animal models, the nebulized IVIg was tested in a human *in vitro* air-liquid interface tissue culture model. The IgG formulation reduced viral infections and maintained the integrity of the epithelial tissue (221). Based on this pre-clinical data, a clinical phase I study with the nebulized IVIg (CSL787, not comprising IgA) recently started in healthy subjects and patients with non-cystic fibrosis bronchiectasis [NCT04643587 (222)].

It remains questionable why the multimeric secretory-like IgA preparations were not able to reproduce the *in vitro* efficacy in these animal models. Perhaps the recombinant SC lost its protective functions *in vivo*. This could also explain why intravenously applied IgM/A/G, which was transported naturally onto mucosa protected mice from infection (160). Nevertheless, the available data show that application of IgA to the lung *via* nebulization is feasible and could open the door for future research in this field. Current alternatives to animal testing could be especially useful to answer the open questions (see below).

#### 4.3.2 Recombinant and engineered IgA antibodies

As outlined above only plasma-derived polyclonal IgA antibodies were used in humans so far; recombinant monoclonal IgA antibodies did not proceed further than experimental or pre-clinical testing. Especially for the production of recombinant IgA antibodies, technical issues were limiting. For example, the purification of IgA requires new capture materials, as the usually for IgG purification used materials do not bind IgA (112, 223). Another issue is the natural ability of IgA to form different multimeric forms, as these require high demands in regard of manufacturability and characterization (e.g. distinction between aggregates) (101). One of the biggest issues are the number of glycosylation sites, because the IgA hinge region is heavily glycosylated, it is problematic to generate consistent glycoforms, as well as to implement proper quality control (101, 112, 120, 224). Furthermore, the complexity of IgA in regard to protein size and glycosylation leads to lower expression yields compared to recombinant IgG (101, 223).

To circumvent the persisting problems with recombinant IgA antibodies, recent antibody engineering strategies successfully improved the manufacturability and the pharmacokinetic properties of IgA. These strategies include modification of the sugar residues (e.g. increased sialylation or removal of N-linked glycosylation sites), addition of an albumin binding domain (which enables recycling by FcRn) or generation of IgG-IgA hybrid antibodies with a similar half-life as IgG1 (27, 112, 120). Furthermore, by using different IgA-engineering approaches and alternative expression systems, the production of IgA multimers was recently successfully established (101, 223).

The idea of therapeutic IgA antibodies gets new attention during COVID-19 pandemic, as a notable high virus neutralization capacity of these multimeric isotypes compared to IgG was shown (225–227). Therefore several studies were published aiming to produce recombinant multimeric IgA antibodies (104, 226, 228, 229). With several advances in manufacturing of recombinant multimeric IgA species, clinical trials seem to be possible (101, 227).

#### 4.3.4 Improvements in research models

There were promising efforts in inducing a protective SIgA immunity due to intranasal vaccination, which is already successfully used against influenza virus (71). Currently intranasal vaccination is in development against SARS-CoV-2 (76, 77), but these efforts are currently hindered by translation from animals to humans (3). This is a commonly known problem in development of respiratory drugs, especially therapeutic antibodies (205, 207, 230, 231).

Although there were successful efforts with transgenic mouse models, more hope can be gained from new alternative strategies with *in silico*, human *ex vivo* tissue-models (e.g. precision-cut lung slices) or 3D-cell culture approaches (4, 7, 230). Due to recent advances in the field of multi-organ chip technologies, which combines the mentioned technologies with microfluidic devices, more systemic and complex responses to the drug can be predicted (230). Although it is clear that animal models will be indispensable in the nearer future and further improvements in the field need to be done (231), these new approaches could improve translational research and could improve our understanding of the behavior of drug candidates, while simultaneously being concordant with the 3R-principles. These approaches may pick up many of the current pitfalls in IgA research and could push the usage of IgA antibodies towards the clinical stage.

## 5 Conclusion

The picture of IgA has changed dramatically in the last decades. There is growing evidence showing complex and potent immune functions of IgA in the context of infection and inflammation (**Figure 1**). Diverse approaches help to understand the dual function of IgA, as protector against invading pathogens and as keeper of immune homeostasis. These dual properties designate IgA as a promising therapeutic agent for the treatment of inflammatory lung diseases, which require both protection against recurrent infections and control of overwhelming inflammation.

Reasoned in several pitfalls, previous efforts in using IgA as a therapeutic antibody are rare. Plasma-derived IgA preparations are currently the only therapeutically used IgA antibodies. Although they contain IgM and IgG antibodies, there is evidence for potent immunomodulatory functions mediated by the IgA component. With further advances in the production of recombinant antibodies, also complex, multimeric IgA molecules may be produced in large scale and with high quality. This could open the door for the wide application of therapeutic IgA preparations.

Targeting the IgA-FcαRI axis on neutrophils recently became a focus in cancer therapy, but this also seems to be a promising strategy in the treatment of inflammatory lung diseases. Both IgA and neutrophils were shown to be important players in lung inflammation. Neutrophils can perform a dual role, because they are elementary for the elimination of invading pathogens, but can also be detrimental and responsible for overwhelming inflammation and tissue damage. IgA and neutrophil dysfunction seems to have a harmful, reinforcing effect on each other in inflammatory lung diseases. This is underpinned by recent data from COVID-19, which show the important role of IgA and NETs in neutrophil-mediated pathology (78, 121, 232). As the IgA-FcαRI axis has a key role in the control of neutrophil function, it could be interesting to target exhausted neutrophils and control the inflammation in lung diseases with therapeutic IgA antibodies. Apart from the effects mediated *via* the IgA-FcαRI-axis it was recently shown that FcαRI is a potent innate receptor that binds bacteria directly and independent of IgA (233). Therefore it could be worth focusing additionally on FcαRI as possible therapeutic target, acting before IgA-mediated responses take place.

New approaches with direct application of IgA to the lung target site or induction of mucosal SIgA response seem to be promising, both in view of the benefits of self-administration and clinical efficacy. Currently, for the clinical success of these approaches the translation of small animal models to humans remains a major issue in respiratory research (7, 230). Future progress in the development of alternative methods could circumvent issues with animal experiments in respiratory research and boost IgA approaches.

After a long time with limited innovations and a series of rather disappointing results, interest in IgA as a therapeutic option has ceased. However, recent advances in the field, justify to reinvestigate the potential of IgA as a therapeutic agent in the treatment of inflammatory lung diseases. Nevertheless, further research is needed to unravel the most suitable form of administration and IgA molecule structure for a successful therapy in these indications.

## Author contributions

The author confirms being the sole contributor of this work and has approved it for publication.
